# Reciprocal Associations between Electronic Media Use and Behavioral Difficulties in Preschoolers

**DOI:** 10.3390/ijerph15040814

**Published:** 2018-04-21

**Authors:** Tanja Poulain, Mandy Vogel, Madlen Neef, Franziska Abicht, Anja Hilbert, Jon Genuneit, Antje Körner, Wieland Kiess

**Affiliations:** 1 LIFE Leipzig Research Center for Civilization Diseases, Leipzig University, Philipp-Rosenthal-Strasse 27, 04103 Leipzig, Germany; mvogel@life.uni-leipzig.de (M.V.); mneef@life.uni-leipzig.de (M.N.); fabicht@life.uni-leipzig.de (F.A.); antje.koerner@medizin.uni-leipzig.de (A.K.); wieland.kiess@medizin.uni-leipzig.de (W.K.); 2 Integrated Research and Treatment Center Adiposity Diseases, Department of Medical Psychology and Medical Sociology, Leipzig University, Philipp-Rosenthal-Strasse 27, 04103 Leipzig, Germany; anja.hilbert@medizin.uni-leipzig.de; 3 Institute of Epidemiology and Medical Biometry, Ulm University, Helmholtzstrasse 22, 89081 Ulm, Germany; jon.genuneit@uni-ulm.de; 4 Department of Women and Child Health, Hospital for Children and Adolescents and Center for Pediatric Research (CPL), Leipzig University, Liebigstrasse 20a, 04103 Leipzig, Germany

**Keywords:** media use, behavioral difficulties, preschool, longitudinal

## Abstract

The use of electronic media has increased substantially and is already observable in young children. The present study explored associations of preschoolers’ use of electronic media with age, gender, and socio-economic status, investigated time trends, and examined reciprocal longitudinal relations between children’s use of electronic media and their behavioral difficulties. The study participants included 527 German two- to six-year-old children whose parents had provided information on their use of electronic media and their behavioral difficulties at two time points, with approximately 12 months between baseline and follow-up. The analyses revealed that older vs. younger children, as well as children from families with a lower vs. higher socio-economic status, were more often reported to use electronic media. Furthermore, the usage of mobile phones increased significantly between 2011 and 2016. Most interestingly, baseline usage of computer/Internet predicted more emotional and conduct problems at follow-up, and baseline usage of mobile phones was associated with more conduct problems and hyperactivity or inattention at follow-up. Peer relationship problems at baseline, on the other hand, increased the likelihood of using computer/Internet and mobile phones at follow-up. The findings indicate that preschoolers’ use of electronic media, especially newer media such as computer/Internet and mobile phones, and their behavioral difficulties are mutually related over time.

## 1. Introduction

The use of electronic media represents a major leisure activity, not only in adolescents [1,2] but also in younger children [3]. The popularity of electronic media has increased substantially among youth today, especially with respect to computers and mobile media [2,4,5]. At the same time, Internet and smartphone addictions, as well as gaming disorders, have gained importance and have become a public health concern worldwide [6,7,8,9]. Internet gaming disorder was even introduced as a “condition for further study” in the Diagnostic and Statistical Manual of Mental Disorders (DSM-5) [10].

Whereas electronic media use by adolescents has been widely studied, less is known about media use by preschool children. The findings of these studies suggest an increase in the use of electronic media as children grow older [11]. Furthermore, children growing up in families with lower socio-economic status (SES) were shown to use more electronic media than children from families with higher SES [11,12,13,14]. While the popularity of different media devices differs between adolescent boys and girls [1,2,15,16], no gender differences have been observed in preschool children [11,14]. 

A frequent use of electronic media at preschool age has been linked to reduced physical fitness and somatic health but also to decreased well-being and psycho-social health [17]. Previous cross-sectional studies revealed that preschool children who spend more time in front of the TV screen have more behavioral difficulties and sleep problems and show a generally lower psycho-social health than children who spend less time in front of the TV [18,19,20,21,22]. There is also evidence for associations between computer gaming and attention deficit disorders in preschool children [23]. Longitudinal studies in this field showed negative relations between TV use at one or three years of age and the development of anti-social behavior and hyperactivity two to four years later [24,25]. Another longitudinal study revealed that higher use of computer games in two- to six-year-old children predicts more emotional problems two years later, whereas higher use of TV predicts lower family functioning [26]. Finally, a longitudinal study in elementary school children in Korea showed that the time spent on voice calls and gaming on mobile phones was associated with an increased risk to show symptoms of attention deficit disorders [27]. These studies suggest an adverse effect of electronic media use on child development. Mechanisms argued to guide these effects are overstimulation of the developing brain, distraction, and displacement of social and physical activities [28]. 

The present study investigated the use of different electronic media in German preschool children. Whereas previous studies mainly focused on preschoolers’ use of TV or video games, here, we looked at the use of traditional as well as newer electronic media (TV/video, game console, computer/Internet, and mobile phone). 

Our first research aim was to assess associations of preschoolers’ media use with age, gender, and SES. We thereby expected to replicate findings of previous studies, i.e., to observe a higher media use in older as compared to younger children and in children growing up in families with a lower versus higher SES, but no differences between boys and girls. 

Another aim of this study was to show how media use by preschoolers changed in the last years. Previous time-trend analyses focused on media use by adolescents until 2010 [2,4,5]. The present data set allowed the investigation of more recent time trends (from 2011 to 2016) for the use of electronic media in preschoolers. We expected to find a similar increase of electronic media use as observed in adolescents [2,4,5]. 

The central research question of the present study was whether reciprocal longitudinal relations between preschoolers’ use of electronic media and their behavioral difficulties (emotional problems, conduct problems, hyperactivity/inattention, and peer relationship problems) would occur (or not). Early psychological and behavioral problems are predictive for later social, academic, and psychological impairment and, therefore, represent key aspects of child development [29,30]. 

Based on previous findings, we hypothesized to find a positive association between preschoolers’ use of electronic media and their amount of behavioral difficulties (regarding individual differences in test scores and the assignments to risk groups) one year later. In contrast to previous prospective studies [24,25,26,27], we also investigated the relationships between preschoolers’ behavioral difficulties and their electronic media use one year later. This design made it possible to reveal longitudinal interdependencies between different aspects of electronic media use and behavioral difficulties. 

## 2. Materials and Methods

### 2.1. Participants

Data were collected between 2011 and 2017 in the LIFE Child study center in Leipzig, Germany. The LIFE Child study is a longitudinal childhood cohort study aiming to investigate healthy child development and the development of civilization diseases [31,32]. Participants are mainly recruited via advertisement at different institutions, such as hospitals and public health centers. The first study visit may take place between 0 and 16 years, and subsequent visits are scheduled every year. The study program consists of several examinations, tests, and questionnaires that are completed by parents (as in the present study) or children themselves. For the present study, data of all two- to six-year-old preschool children who had participated at two time points, a baseline visit and a follow-up visit, were analyzed. The baseline visit took place between 2011 and 2016. The follow-up visit took place approximately 12 months (M = 12.23 months, SD = 1.05 months) after the baseline visit, i.e., between 2012 and 2017. The drop-out rate between baseline and follow-up was 25%. The final sample consisted of 527 preschool children (272 male, 255 female). The mean age was 3.81 (SD = 0.89) at baseline and 4.83 (SD = 0.90) at follow-up. The SES of the participating families was reflected by the so-called Winkler index, an index that considers the education (graduation and professional education) and occupational status of mothers and fathers as well as the monthly family net income [33,34]. This index ranges between 3 and 21, with higher scores indicating higher SES. Based on the appropriate classification algorithm [34], 9% of the participants were assigned to the lower social milieu (index 3–8.4), 59% to the middle social milieu (index 8.5–15.4), and 32% to the high social milieu (index 15.5–21). 

Informed written consent was provided by all parents before the inclusion of their children in the study. The study was conducted in accordance with the Declaration of Helsinki, and the study protocol was approved by the Ethics Committee of the University of Leipzig (Reg. No. 264-10-19042010). 

### 2.2. Instruments

Children’s use of different electronic media was assessed by a questionnaire that was originally designed for the use in the KiGGS study (German Health Interview and Examination Survey for Children and Adolescents), a population-based nationwide survey on the health of German children [1]. In this questionnaire, parents are asked to judge how many hours per day their children usually spend using TV/video, game consoles, computer/Internet, and mobile phones (never, approximately 30 min, between 1 and 2 h, between 3 and 4 h, longer than 4 h). Given that the participating children generally spent little time with the different media, only non-usage (represented by answer category “never”) and usage (represented by the other answer categories) were distinguished for further analysis. 

Behavioral difficulties of children were assessed using the parent version of the Strengths and Difficulties Questionnaire (SDQ), a standard instrument measuring behavior difficulties and strengths of children and adolescents [35,36]. The five scales of the SDQ assess children’s emotional problems, conduct problems, hyperactivity/inattention, peer relationship problems, and pro-social behavior. Each scale consists of five questions. Answers are given on a three-grade scale (0 = not true, 1 = somewhat true, 2 = certainly true). For the present analysis, the problem scales (all scales except pro-social behavior) as well as the total difficulties score (sum score of all problem scales) were investigated and analyzed in two different ways. First, we looked at differences in each score (ranging between 0 and 10 in the single problems scales and between 0 and 40 in the total difficulties score). This strategy allowed an assessment of individual differences spanning the whole range of possible outcomes. However, it provided no information on whether behavior has to be judged as normal or risky. Therefore, a second step of analysis consisted of assigning children to either a normal or a risk group. The categorization was based on scale-specific cut-off scores identified in a large sample of German children and adolescents [36]. All scores as high as or lower than these reference cut-off scores indicate that a behavioral pattern can be considered as “normal”. In contrast, all scores above the cut-off scores indicate that a form of behavior has to be considered as “risky.” This categorization has already been applied in other studies [37,38]. The cut-off scores separating normal from risky behaviors are 3 for the emotional problems, conduct problems, and peer relationship problems scale, 5 for the hyperactivity/inattention scale, and 12 for the total difficulties score of the SDQ [36].

### 2.3. Statistical Analysis

All analyses were conducted using R, version 3.3.2 (R Foundation for Statistical Computing, Vienna, Austria). Multiple logistic regressions with either the usage of TV/video, game consoles, computer/Internet, or mobile phones at baseline as the dependent variable and the usage of the other three media at baseline as independent variables were applied to reveal relations between different forms of electronic media usage. 

Associations of children’s use of electronic media with socio-demographic aspects as well as the year of data acquisition were assessed by multiple logistic regression analyses with age, gender, SES (Winkler index), and year of data acquisition as independent variables and the usage of the different electronic media as dependent variables. These analyses were based on data collected at baseline.

In order to assess associations between children’s use of electronic media at baseline and their behavioral difficulties at follow-up, two analyses were performed. In a first analysis (multiple linear regression), the usages of the different electronic media at baseline (exposure) were associated with the individual scores in the different problems scales of the SDQ at follow-up (outcome). A second analysis (multiple logistic regression) comprised the same exposure variables, but assigned the risk group for each problem scale as outcome. All associations were adjusted for age, gender, SES, year of data acquisition, and the outcome at baseline. Therefore, the analyses indicate if electronic media use at baseline is associated with the evolution of behavioral difficulties between baseline and follow-up.

To test the reverse associations, i.e., associations between behavioral difficulties at baseline and electronic media use at follow-up, multiple logistic regressions with the baseline scores in the single problem scales of the SDQ as exposure and the usages of the single electronic media at follow-up as outcomes were performed. Similarly, the associations were adjusted for age, gender, SES, year of data acquisition, and outcome at baseline. 

In order to investigate if associations between media use and behavioral difficulties differ depending on age, gender, and SES, every single association was checked for interactions with these control variables. Preconditions for including an interaction in the final model were the significance of the interaction (*p* < 0.05) and preservation of the model quality. The inclusion of an interaction was considered to preserve the quality of a statistical model if it did not cause severe inflation of variance (indicated by a variance inflation factor <5). 

## 3. Results

### 3.1. Time Spent in Using Different Electronic Media

The media usage by the participating preschoolers is summarized in Table 1. As can be seen, only a few children (between 3 and 11%) were reported to use game consoles, computer/Internet, or mobile phones at baseline and follow-up. In contrast, approximately 80% watched TV/video on a daily basis. Thereby, most of the children (56% at baseline and 58% at follow-up) were reported to watch TV/video for approximately 30 min per day. However, about 20% of children watched TV for more than one hour per day. The likelihood of using computer/Internet was significantly increased by the use of TV/video (OR = 8.72, *p* < 0.05) and by the use of game consoles (OR = 8.11, *p* < 0.001). No other significant associations between the usages of the different electronic media could be shown (all *p* > 0.05). 

### 3.2. Behavioral Difficulties in the Present Sample

The behavioral difficulties in the present study sample are displayed in Table 1. The average scores in the single problem scales ranged between 1.21 (peer relationship problems scale at follow-up) and 3.99 (hyperactivity/inattention scale at baseline). For the majority of children, the total difficulties score lay in the normal range. However, approximately 20% of children were assigned to the total difficulties risk group (19% at baseline, 22% at follow-up). Looking at the single problem scales, the hyperactivity/inattention risk group was assigned most frequently (20% at baseline and follow-up) and the peer relationship problems risk group least frequently (9% at baseline, 8% at follow-up).

### 3.3. Associations of Media Usage with Age, Gender, SES, and Year of Data Acquisition

**Figure 1:**
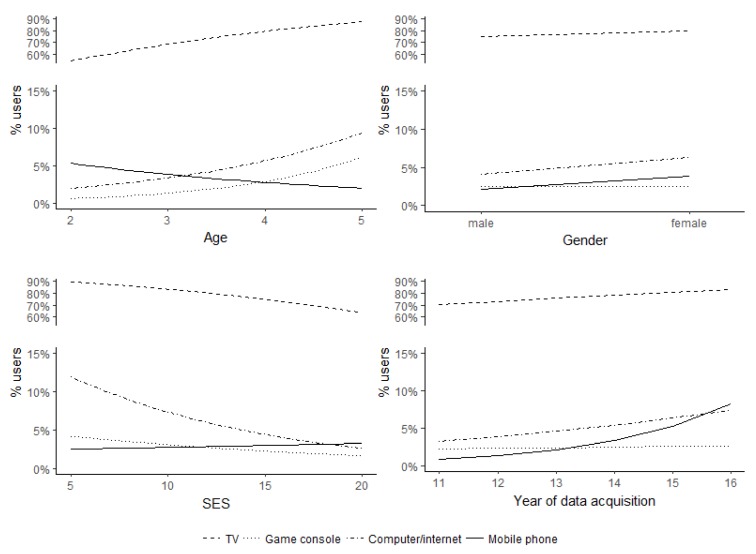
Effect plots for the associations of age, gender, socio-economic status (SES), and year of data acquisition with the use of different electronic media. The y-axis was modified due to differences in the usages of the different electronic media.

The relationships between participants’ use of electronic media and age, gender, SES, and the year of data acquisition are displayed in Figure 1. As revealed by the statistical analyses, higher child age significantly heightened the likelihood of watching TV/video (OR = 1.79, *p* < 0.001), using game consoles (OR = 2.24, *p* < 0.01), and using computer/Internet (OR = 1.73, *p* < 0.05). The usage of mobile phones, in contrast, showed no significant association with child age (OR = 0.71, *p* = 0.29). Gender was not associated with the use of electronic media (all *p* > 0.05). Higher SES significantly decreased the likelihood of watching TV/video (OR = 0.90, *p* < 0.01) and of using computer/Internet (OR = 0.90, *p* < 0.05), but was not associated with the use of game consoles and mobile phones (all *p* > 0.05). Regarding time trends, a significant association between the use of mobile phones and the year of data acquisition (OR = 1.62, *p* < 0.05) indicated an increase in the usage of mobile phones from 2011 to 2016. The likelihood of watching TV/video and of using game consoles or computer/Internet, however, did not change significantly between 2011 and 2016 (all *p* > 0.05).

### 3.4. Reciprocal Longitudinal Associations between the Use of Electronic Media and Behavioral Difficulties

Table 2 summarizes the associations between children’s use of different electronic media at baseline and their behavioral difficulties at follow-up. With respect to individual differences in the single scores of the SDQ (see Table 2a), the use of computer/Internet and mobile phones at baseline was significantly associated with higher total difficulties scores at follow-up (b = 1.76 and 2.21, *p* < 0.01, respectively). Concerning the single problems scales of the SDQ, the use of computer/Internet at baseline was associated with higher scores in the emotional problems scale (b = 0.57, *p* < 0.05) and in the conduct problems scale at follow-up (b = 0.65, *p* < 0.01). The baseline use of mobile phones was associated with higher scores in the conduct problems scale (b = 0.55, *p* < 0.05) and in the hyperactivity/inattention scale at follow-up (b = 1.10, *p* < 0.01). 

With regard to the assignment of children to the risk group of behavioral difficulties (see Table 2b), the use of mobile phones was associated with an increased likelihood to be assigned to the total difficulties risk group (OR = 3.25, *p* < 0.05) and the hyperactivity/inattention risk group (OR = 3.36, *p* < 0.05). Furthermore, the baseline use of computer/Internet was associated with an elevated likelihood to belong to the total difficulties risk group at follow-up (OR = 3.03, *p* > 0.05). 

The reverse associations, i.e., the associations between children’s behavioral difficulties at baseline and their use of electronic media at follow-up, are displayed in Table 3. As can be seen, higher baseline scores in the peer relationship problems scale significantly heightened the likelihood of using computer/Internet (OR = 1.28, *p* < 0.05) and mobile phones at follow-up (OR = 1.58, *p* < 0.001). 

### 3.5. Differences in Associations between Media Usage and Behavioral Difficulties Depending on Child Age, Gender, and SES

All statistical models presented in Table 2 and Table 3 were checked for interactions between each independent variable and age, gender, or SES. However, interactions did not reach significance or limited the quality of the model by causing severe inflation of variance. Therefore, the associations reported in Table 2 and Table 3 can be assumed to not differ depending on child age, gender, or SES.

## 4. Discussion

The present study investigated the use of electronic media in a sample of two- to six-year-old German preschool children. It assessed associations of electronic media use with socio-demographic parameters and year of data acquisition, as well as reciprocal longitudinal associations with behavioral difficulties in different domains. 

### 4.1. Use of Electronic Media

In the present sample, the use of game consoles, computer/Internet, or mobile phones was low. However, nearly 80% of children were reported to watch TV or videos on a daily basis. Approximately 20% watched TV for more than one hour per day and, therefore, exceeded the recommendations to limit daily screen times to a maximum of 30 min [39]. Interestingly, children who watched TV/video or used game consoles were more likely to use computer/Internet than children who did not use these media. This finding shows that children usually use several types of media. This dynamic might be explained by children’s or parents’ general attitudes towards media or sedentary behaviors in general. Children who are interested in using one medium may also be interested in using other media. Similarly, parents who support the usage of one medium may also allow the purchase or usage of other media.

As already shown in other studies [11], the use of electronic media (except mobile phones) increased with child age. Since the media use by preschoolers is mainly controlled by their parents, this increase might reflect parents’ beliefs in greater suitability and comprehensibility of electronic media for older versus younger children. Whereas the use of mobile phones was not associated with child age, it increased significantly between 2011 and 2016. This finding indicates that the increased usage of newer media, which has already been shown in older children and adolescents [2,4,5], is also observable in younger children. Given the potential negative impact of early media use on child development, this finding is alarming and underlines the importance to support children and parents in using electronic media in a responsible way.

Our results furthermore revealed that children from families with a lower SES were more often reported to watch TV/video and to use computer/Internet than children from families with a higher SES. This finding is in line with previous studies showing longer media usage times in socially disadvantaged families [11,12,13,14] and indicates that programs on how to use electronic media or to limit children’s screen time might be especially helpful in lower social milieus. 

As already shown in other studies [11,14], preschoolers’ electronic media usage did not differ between girls and boys. Gender differences might appear only in later childhood and adolescence [1,2,15,16] when parental control decreases and individual interests of children develop. 

### 4.2. Behavioral Difficulties

Regarding the behavioral difficulties in the present sample, the average scores in the SDQ scales were comparable to scores reported for 3- to 6-year-old children participating in another large German study (KiGGS) [37]. This result indicates that our sample was representative with respect to behavioral difficulties, i.e., it did not reflect a mentally very healthy nor very unhealthy sample. 

In the KiGGS study, the proportions of children assigned to the normal or risk group of behavioral difficulties were not reported for single age groups (e.g., 3- to 6-year-old children), but only for the whole study sample of 3- to 17-year-old children. Therefore, a comparison of the prevalence of risky behaviors in the present versus the KiGGS study is difficult. Compared to the whole sample of 3- to 17-year-olds, the proportions of risky behaviors observed in the present study were higher, at least concerning conduct problems and hyperactivity/inattention. However, these behavior difficulties have been shown to decrease as children grow older [36,37]. Therefore, the differences between the higher scores in the present versus the KiGGS study probably reflect differences in the age of the two study samples rather than differences in hyperactivity/inattention and conduct problems. 

### 4.3. Reciprocal Associations between Electronic Media Usage and Behavioral Difficulties

Concerning relationships between children’s use of electronic media and their later behavioral difficulties in different domains, the analyses revealed that preschoolers who used computer/Internet or mobile phones at their first study visit showed more behavioral difficulties one year later and were more often assigned to the risk group of behavioral difficulties than children who did not use these media at their first study visit. As the associations were controlled for behavioral difficulties at baseline, these findings indicate an increase in behavior difficulties between first and second study visit in children using computer/Internet or mobile phones. 

Looking at the single domains of behavioral difficulties, children who used computer/Internet showed more conduct and emotional problems than children who did not use these media. This finding is in line with another longitudinal study in which the use of computer games was associated with emotional problems two years later [26]. Children who used mobile phones showed more conduct problems and more signs of hyperactivity/inattention than children who did not use mobile phones. The baseline usage of mobile phones was also associated with an increased likelihood of belonging to the hyperactivity/inattention risk group one year later, i.e., of showing a level of hyperactivity/inattention beyond the normal range. This result strengthens the findings of a longitudinal study in elementary school children showing associations between mobile phone use (for gaming and voice calls) and symptoms of attention deficit disorders [27]. In contrast to the usage of computers and mobile phones, television viewing showed no significant associations with later behavioral difficulties. This finding contradicts other longitudinal studies at preschool age showing relations between a prolonged TV usage and later hyperactivity and aggressive behavior [24,25]. However, these studies have been conducted several years ago and did neither consider newer media nor control for behavioral difficulties at baseline. The present findings suggest that at this time, it is not the usage of TV, but rather the use of newer media that relate to the evolution of behavioral difficulties in preschool children. Possible reasons for this relation are an overstimulation of the developing brain, distraction, and displacement of other activities, e.g., social and physical activities [28]. Due to small screens, Internet access, and rapid flow of information, overstimulation and distraction might be especially pronounced during the use of computers and mobile phones. Future studies might investigate the reason why young children use computers and mobile phones (e.g., gaming, watching videos) and if different media contents and using modes (e.g., active versus passive) show different effects on child development and health. 

In this study, we also investigated associations between baseline behavioral difficulties and later media usage. This analysis revealed that children who were reported to have more peer relationship problems at baseline were more likely to use computer/Internet and mobile phones one year later than children showing fewer peer relationship problems. Children who are less integrated and have fewer social contacts might be prone to other, less social, leisure activities such as electronic media usage. They might, furthermore, spend more time with their parents or (older) siblings and, therefore, use the media used by these reference persons, e.g., computers and mobile phones. 

Overall, these results suggest that media use and behavioral difficulties are interrelated and that the effects of both might mutually reinforce each other to impact child development negatively. Therefore, the early use of mobile phones and computers as well as early signs of behavioral difficulties should be taken seriously in order to maintain children’s well-being.

### 4.4. Limitations

The longitudinal design and consideration of traditional as well as newer media in one statistical model represent the strengths of this study. However, some limitations have to be mentioned. First of all, all measures were based on parental reports. Parents might not disclose any behavioral difficulties in their children and tend to underestimate the actual electronic media usage of their children. The mere distinction between usage and non-usage of electronic media and the lacking assessment of media content and purpose represent further limitations of this study. Future research might try to apply more detailed (e.g., the distinction between week and weekend, and between offline and online usage) and more objective measures of media use. 

Another limitation is that the cut-off scores used to assign children to SDQ risk groups were based on data collected in older (6- to 16-year-old) children. However, to our knowledge, there exist no normative data for younger German children. 

Finally, we acknowledge a limited representativity in the present study sample, especially with regard to participants’ SES. Even if SES was controlled for in all statistical models, the limited representativity might reduce the ability of the study results to be generalized. 

## 5. Conclusions

The present study shows an increase in preschoolers’ use of newer electronic media in recent years and reveals a significant association of the use of these media with later behavioral difficulties. At the same time, the use of electronic media was found to be associated with earlier behavioral difficulties in children. It can be concluded that preschoolers’ electronic media use and psychological health are mutually related over time. In order to maintain healthy child development, early media use and behavioral difficulties should be taken seriously and treated adequately. Future studies might investigate which mechanisms trigger the associations between media use and behavioral difficulties and how young children and their parents, especially in lower social milieus, can be supported in using electronic media appropriately. 

## Figures and Tables

**Table 1:** Electronic media consumption and behavioral difficulties in the present sample (N = 537).

Electronic Media Usage			Baseline	Follow-Up
		
TV/video	never	N (%)	127 (24%)	99 (19%)
	30 min	N (%)	296 (56%)	305 (58%)
	1–2 h/day	N (%)	98 (19%)	123 (23%)
	3–4 h/day	N (%)	3 (1%)	0 (0%)
	>4 h/day	N (%)	0 (0%)	0 (0%)
Game console	never	N (%)	510 (97%)	489 (93%)
	30 min	N (%)	12 (2%)	34 (6%)
	1–2 h/day	N (%)	4 (1%)	4 (1%)
	3–4 h/day	N (%)	0 (0%)	0 (0%)
	>4 h/day	N (%)	1 (0%)	0 (0%)
Computer/Internet	never	N (%)	495 (94%)	468 (89%)
	30 min	N (%)	26 (5%)	55 (10%)
	1–2 h/day	N (%)	5 (1%)	4 (1%)
	3–4 h/day	N (%)	1 (0%)	0 (0%)
	>4 h/day	N (%)	0 (0%)	0 (0%)
Mobile phone	never	N (%)	506 (96%)	499 (95%)
	30 min	N (%)	21 (4%)	27 (5%)
	1–2 h/day	N (%)	0 (0%)	1 (0%)
	3–4 h/day	N (%)	0 (0%)	0 (0%)
	>4 h/day	N (%)	0 (0%)	0 (0%)
Behavioral difficulties				
Emotional problems	Score	M (SD)	1.60 (1.58)	1.76 (1.81)
	Normal group	N (%)	464 (88%)	437 (83%)
	Risk group	N (%)	63 (12%)	89 (17%)
Conduct problems	Score	M (SD)	2.25 (1.47)	2.15 (1.55)
	Normal group	N (%)	432 (82%)	432 (81%)
	Risk group	N (%)	95 (18%)	95 (19%)
Hyperactivity/inattention	Score	M (SD)	3.99 (2.24)	3.84 (2.40)
	Normal group	N (%)	423 (80%)	420 (80%)
	Risk group	N (%)	104 (20%)	106 (20%)
Peer relationship problems	Score	M (SD)	1.32 (1.48)	1.21 (1.43)
	Normal group	N (%)	480 (91%)	484 (92%)
	Risk group	N (%)	47 (9%)	42 (8%)
Total difficulties	Score	M (SD)	9.16 (4.52)	8.96 (4.97)
	Normal group	N (%)	426 (81%)	412 (78%)
	Risk group	N (%)	101 (19%)	114 (22%)

**Table 2:** Association of electronic media usage at baseline with behavioral difficulties at follow-up (N = 537) ^a^.

Table 2a. Behavioral Difficulties as Individual Differences in SDQ Scores						Table 2b. Behavioral Difficulties as Assignment to SDQ Risk Groups				
Exposure (Baseline)	Outcome (Follow-Up)					Outcome (Follow-Up)				
	SDQ Problem Score at Follow-Up					Classification as Risk Group Based on SDQ Scores				
	Total Difficulties	Emotional Problems	Conduct Problems	Hyperactivity Inattention	Peer Relationship Problems	Total Difficulties	Emotional Problems	Conduct Problems	Hyperactivity Inattention	Peer Relationship Problems
	b (95% CI)	b (95% CI)	b (95% CI)	b (95% CI)	b (95% CI)	OR (95% CI)	OR (95% CI)	OR (95% CI)	OR (95% CI)	OR (95% CI)
TV/video ^b^	−0.15 (−0.89–0.59)	−0.06 (−0.38–0.25)	−0.19 (−0.45–0.07)	0.20 (−0.17–0.57)	−0.05 (−0.29–0.20)	0.82 (0.46–1.46)	0.63 (0.35–1.12)	1.08 (0.59–1.97)	1.23 (0.65–2.33)	0.91 (0.39–2.12)
Game console ^b^	−0.71 (−2.50–1.08)	−0.28 (−1.04–0.48)	−0.40 (−1.02–0.21)	0.26 (−0.62–1.15)	−0.31 (−0.89–0.28)	1.64 (0.49–5.52)	0.47 (0.09–2.34)	1.11 (0.29–4.31)	1.81 (0.50–6.45)	0.37 (0.03–4.19)
Computer/Internet ^b^	1.76 (0.43–3.09) **	0.57 (0.00–1.13) *	0.65 (0.19–1.11) **	0.49 (−0.17–1.15)	0.19 (−0.25–0.63)	3.03 (1.26–7.28) *	0.98 (0.35–2.77)	1.63 (0.63–4.16)	1.73 (0.67–4.47)	1.83 (0.52–6.44)
Mobile phone ^b^	2.21 (0.62–3.79) **	0.35 (−0.32–1.02)	0.55 (0.01–1.10) *	1.10 (0.31–1.88) **	0.16 (−0.36–0.68)	3.25 (1.19–8.92) *	0.68 (0.17–2.68)	1.52 (0.51–4.59)	3.36 (1.21–9.34) *	2.45 (0.49–12.11)

^a^ All associations are adjusted for age, gender, socio-economic status (SES), year of data acquisition, the other predictors, and the outcome at baseline; ^b^ Reference = non-usage; b = regression coefficient, non-standardized. OR = Odds ratio; ** *p* < 0.01, * *p* < 0.05.

**Table 3:** Association of behavioral difficulties at baseline with electronic media usage at follow-up (N = 537) ^a^.

Exposure (Baseline)	Outcome (Follow-Up)			
	Usage of Screen-Based Media			
	TV/Video	Game Console	Computer/Internet	Mobile Phone
	OR (95% CI)	OR (95% CI)	OR (95% CI)	OR (95% CI)
Emotional problems	0.96 (0.80–1.16)	1.10 (0.87–1.39)	0.96 (0.78–1.18)	0.87 (0.66–1.15)
Conduct problems	0.99 (0.80–1.23)	1.11 (0.84–1.46)	0.91 (0.72–1.15)	0.90 (0.64–1.25)
Hyperactivity/inattention	1.09 (0.95–1.24)	0.97 (0.80–1.17)	1.03 (0.88–1.20)	0.93 (0.74–1.15)
Peer relationship problems	0.92 (0.76–1.12)	0.83 (0.62–1.10)	1.28 (1.04–1.58) *	1.58 (1.21–2.06) ***

^a^ All associations are adjusted for age, gender, socio-economic status (SES), year of data acquisition, the other predictors, and the outcome at baseline; *** *p* < 0.001, * *p* < 0.05.
